# mHealthApps: A Repository and Database of Mobile Health Apps

**DOI:** 10.2196/mhealth.4026

**Published:** 2015-03-18

**Authors:** Wenlong Xu, Yin Liu

**Affiliations:** ^1^Department of Neurobiology and AnatomyUniversity of Texas Health Science Center at HoustonHouston, TXUnited States; ^2^University of Texas Graduate School of Biomedical ScienceHouston, TXUnited States

**Keywords:** mobile healtlh, app repository, app database

## Abstract

**Background:**

The market of mobile health (mHealth) apps has rapidly evolved in the past decade. With more than 100,000 mHealth apps currently available, there is no centralized resource that collects information on these health-related apps for researchers in this field to effectively evaluate the strength and weakness of these apps.

**Objective:**

The objective of this study was to create a centralized mHealth app repository. We expect the analysis of information in this repository to provide insights for future mHealth research developments.

**Methods:**

We focused on apps from the two most established app stores, the Apple App Store and the Google Play Store. We extracted detailed information of each health-related app from these two app stores via our python crawling program, and then stored the information in both a user-friendly array format and a standard JavaScript Object Notation (JSON) format.

**Results:**

We have developed a centralized resource that provides detailed information of more than 60,000 health-related apps from the Apple App Store and the Google Play Store. Using this information resource, we analyzed thousands of apps systematically and provide an overview of the trends for mHealth apps.

**Conclusions:**

This unique database allows the meta-analysis of health-related apps and provides guidance for research designs of future apps in the mHealth field.

##  Introduction

With the constant expansion of mobile health (mHealth) in the past few years, the market of mobile apps related to health is rapidly evolving, making countless new mobile technologies potentially available to the health care system. According to a new report (May 2014) generated by the Research2Guidance firm [1], there are more than 100,000 apps falling into the health, fitness, or medical categories, which doubles the market size of that in two and a half years ago. Recently, there have been a number of studies in the field, including the development of a mHealth behavior change system [2], the creation of a food database [3], and a collaborative effort aiming to integrate apps platform, research data repository, and patient summarization [4]. However, there is still a lack of systematic research on the impact of the mHealth apps on health outcomes.

Currently, most research in this field often investigates the apps individually, either by searching the apps from app stores, or by manually installing each individual app on smartphones or tablets one by one [5-8] to get the detailed information of each app. For example, Chomutare et al manually installed 488 diabetes related apps to review their features [5]. Sama et al manually installed around 400 apps to evaluate existing mHealth app tools [6]. Due to the difference in health conditions and app specialization, Tomlinson et al suggested an open mHealth architecture-based platform to facilitate scalable and sustainable health information systems [7]. While the app stores provide a wealth of information including the prices and customer reviews for apps [9], there is not a centralized resource that collects information of all health-related apps for researchers to systematically evaluate the apps regarding their effectiveness and health outcome. In this study, we aim to obtain a comprehensive view on the mHealth apps by creating an app repository. We expect the analysis of apps in this repository can provide insights for future mHealth research developments.

## Methods

### Repository Based on the Apple App Store

Since the Apple App Store (AppStore) is the major representative in the market, we first created an app repository based on all the health related apps from the AppStore. The list of apps was crawled from the Apple iTunes Web pages [10], including the pages for the Health & Fitness [11] and the Medical [12] subcategories. Then using our own crawling program, we extracted detailed information of each app via the iTunes Search app program interface (API) [13]. We noticed the results from our data extraction step are in the JavaScript Object Notation (JSON) [14] format. For the convenience of researchers, we transferred the files from JSON format to tab-delimited text files encoded with “utf8mb4” (flat files with array format), so that researchers can directly import these files to Excel or another program for ease of analysis. In the text files, each row corresponds to an app with 39 features, including the app unique identity (ID), app name, description, user rating count, average user rating, etc. Table 1 lists all the 39 features along with their annotations.

### Repository Based on the Google Play Store

Since the Google Play Store (GooglePlay) is now the biggest app store in the market, we also created an app repository based on the information of all popular health-related apps from the GooglePlay. The list of apps was crawled from the GooglePlay Web pages [15], including the pages for the HEALTH_AND_FITNESS [16] and MEDCIAL [17]. We then extracted the detailed information of each popular app using the python HyperText Markup Language parsing tool via the Google Play Search API. For researchers’ convenience, we provided both the JSON format and tab-delimited text files as well. In the text files, each row corresponds to an app with 27 features (Table 2), including the app unique ID, app name, description, user rating count, average user rating, etc. Files in both formats (JSON and tab-delimited) can be obtained from the repository website [18].

**Table 1:** The list of 39 features for each app in the AppStore.

Feature	Annotation
trackId	Unique app ID
artistId	Developer ID
artistName	Name of the developer
artistViewUrl	The URL for the developer
artworkUrl100	The URL for the artwork in 100*100 pixels
artworkUrl512	The URL for the artwork in 512*512 pixels
artworkUrl60	The URL for the artwork in 60*60 pixels
averageUserRating	Average of user ratings
averageUserRatingForCurrentVersion	Average of user ratings for current version
bundleId	Bundle ID
contentAdvisoryRating	Content ratings by content advisor
currency	Currency
description	Description of the app
features	Features
fileSizeBytes	File size in bytes
formattedPrice	Price in currency format
genreIds	Categories IDs
genres	Categories
ipadScreenshotUrls	The URLs for the iPad screenshot
isGameCenterEnabled	Whether it is game center enabled
kind	The kind of content
languageCodesISO2A	Language codes ISO2A
price	Price
primaryGenreId	Primary category ID
primaryGenreName	Primary category name
releaseDate	Release date
releaseNotes	Release notes
screenshotUrls	The URLs for screenshot
sellerName	Seller name
sellerUrl	The URL for the seller
supportedDevices	Supported devices
trackCensoredName	Name (censored)
trackContentRating	Content rating
trackName	App name
trackViewUrl	The URL for the app
userRatingCount	The number of user ratings
userRatingCountForCurrentVersion	The number of user ratings for current version
version	Version number
wrapperType	The name of object

**Table 2:** The list of 27 features for each app in the GooglePlay.

Features	Annotation
trackId	Unique app ID
artworkUrl	The URL for the artwork
averageUserRating	Average of user ratings
badge	Developer badge
category	Category
contentRating	Content rating
description	Description of the app
developerEmail	Developer email address
developerId	Developer ID
developerName	Name of the developer
developerPrivacy	The link to the developer privacy notation
developerWebsite	Developer website
fileSize	File size
formattedPrice	Price in currency format
inAppPurchase	Whether it is in app purchase or not
installs	Number of installations
price	Price
releaseNotes	Release notes
requiresAndroid	Android OS requirement
screenshotUrls	The URLs for screenshot
screenshotVideoUrls	The URLs for video screenshot
trackName	App name
trackViewUrl	The URL for the app
updated	Update date
userRatingCount	The number of user ratings
userRatingCountDistribution	The numbers of ratings with 5, 4, 3, 2, or 1 stars
version	Version number

## Results

### Apps From Apple App Store

In the US market, there are 74,211 apps listed in the Apple iTunes Health & Fitness and Medical subcategories as of December 4, 2014. By removing duplicated entries, we obtained 62,621 totally unique apps in these two subcategories. We note the category of each app is defined by the app’s owner (developer or seller) and approved by Apple’s customer service, so the app categorization was done in the server side (API) and was used directly as our app selection criteria. The primary categories of some apps are neither Health & Fitness nor Medical, but others, such as Lifestyle, Education, Sports, Food & Drink, or Games. To reduce the ambiguity, we only included the 47,883 apps with either Health & Fitness or Medical as their primary category in our app repository. In addition to the US market, this repository contains the information of mHealth apps from the AppStore distributed in four other countries with the most established Internet markets [19]: (1) China (CN), (2) Japan (JP), (3) Brazil (BR), and (4) Russia (RU). There are 27,157 and 21,607 unique apps in the categories of Health & Fitness and Medical from the top five countries of the AppStore, respectively, leading to 48,764 totally unique health-related apps from the top five countries. In both categories, there are slightly more apps available in the United States than in any of the other four countries (Table 3). Overall, more than 98.19% (47,883/48,764) of these unique apps are available in the United States.

**Table 3:** The number of apps in different stores and regions.

Store_region_category	Apps^a^	Free apps	% of free apps	Sum of user ratings^b^	Sum of user ratings (free)^c^	% of user ratings (free apps)
AppStore_BR_Health&Fitness	25,931	16,761	65	79,738	60,924	76
AppStore_BR_Medical	20,047	13,313	66	24,169	18,074	75
AppStore_CN_Health&Fitness	25,845	16,732	65	164,314	137,011	83
AppStore_CN_Medical	19,857	13,173	66	14,765	12,128	82
AppStore_JP_Health&Fitness	25,962	16,809	65	204,012	141,292	69
AppStore_JP_Medical	19,961	13,250	66	21,008	16,426	78
AppStore_RU_Health&Fitness	25,926	16,774	65	139,488	96,348	69
AppStore_RU_Medical	19,912	13,198	66	19,736	15,679	79
AppStore_US_Health&Fitness	26,762	17,521	65	3,596,338	2,877,808	80
AppStore_US_Medical	21,121	14,357	68	866,582	671,408	77
AppStore_Top5Regions_Health&Fitness	27,157	17,813	66	4,183,890	3,313,383	79
AppStore_Top5Regions_Medical	21,607	14,729	68	946,260	733,715	78
GooglePlay_US_Health&Fitness	6894	5155	75	10,921,244	10,446,157	96
GooglePlay_US_Medical	5378	3180	59	900,476	852,068	95

^a^ Apps, the total number of apps in each specified combination of store, region, and category.

^b^ Sum of user ratings, the total number of ratings received from app users.

^c^ Sum of user ratings, free, the total number of ratings received for free apps.

### Apps From Google Play Store

The repository also contains information of the most popular apps from the GooglePlay in the United States. For the GooglePlay, the Web pages only list the most popular or the newest released apps in each category based on their release dates and daily user usage. Since the GooglePlay Web pages are updated daily, to get a comprehensive list of all the apps, we collected the app IDs available on the GooglePlay with our crawling program every day from July 24 to December 6, 2014, and combined the results to get a list of 14,817 unique app IDs. We then excluded the inactive apps that are no longer available on the GooglePlay. In addition, as we did for the AppStore, we also excluded the apps with their primary category other than HEALTH_AND_FITNESS or MEDICAL. Finally, we obtained a list of 12,272 totally unique apps, including 6894 and 5378 apps in the subcategories of HEALTH_AND_FITNESS and MEDICAL, respectively. Table 3 gives the total number of apps and the total number of user ratings received in each category. Considering the fact that the GooglePlay apps in our repository are among the most popular ones, and GooglePlay represents the biggest app store now, it is not surprising to see that the number of user ratings received for the Health & Fitness apps in GooglePlay is more than two times higher than the sum of user ratings collected from the top five countries for the AppStore apps in the same category.

### Price Factors and App Release Trend

According to Table 3, we can deduce that the average number of user ratings received per app in the Health & Fitness category is significantly higher than that in the Medical category, regardless of app stores. Because the number of user ratings reflects the popularity of the app, this comparison result indicates apps in the Health & Fitness category are more popular than those in the Medical category. We further investigated the effect of app prices on their popularity among users. Overall, a majority of mHealth apps are free, especially in the GooglePlay, as high as 74.78% (5155/6894) of apps in the category of Health& Fitness are free apps. Based on Table 3, we can see that the average number of user ratings per free app is always higher than that for a nonfree app. In addition, if we use the average number of user ratings per app as a measure for app popularity, the significantly high percentage of user ratings provided by GooglePlay free app users (95.57%, 11,298,225/11,821,720) suggests the GooglePlay users prefer free apps, compared to the AppStore users.

**Figure 1:**
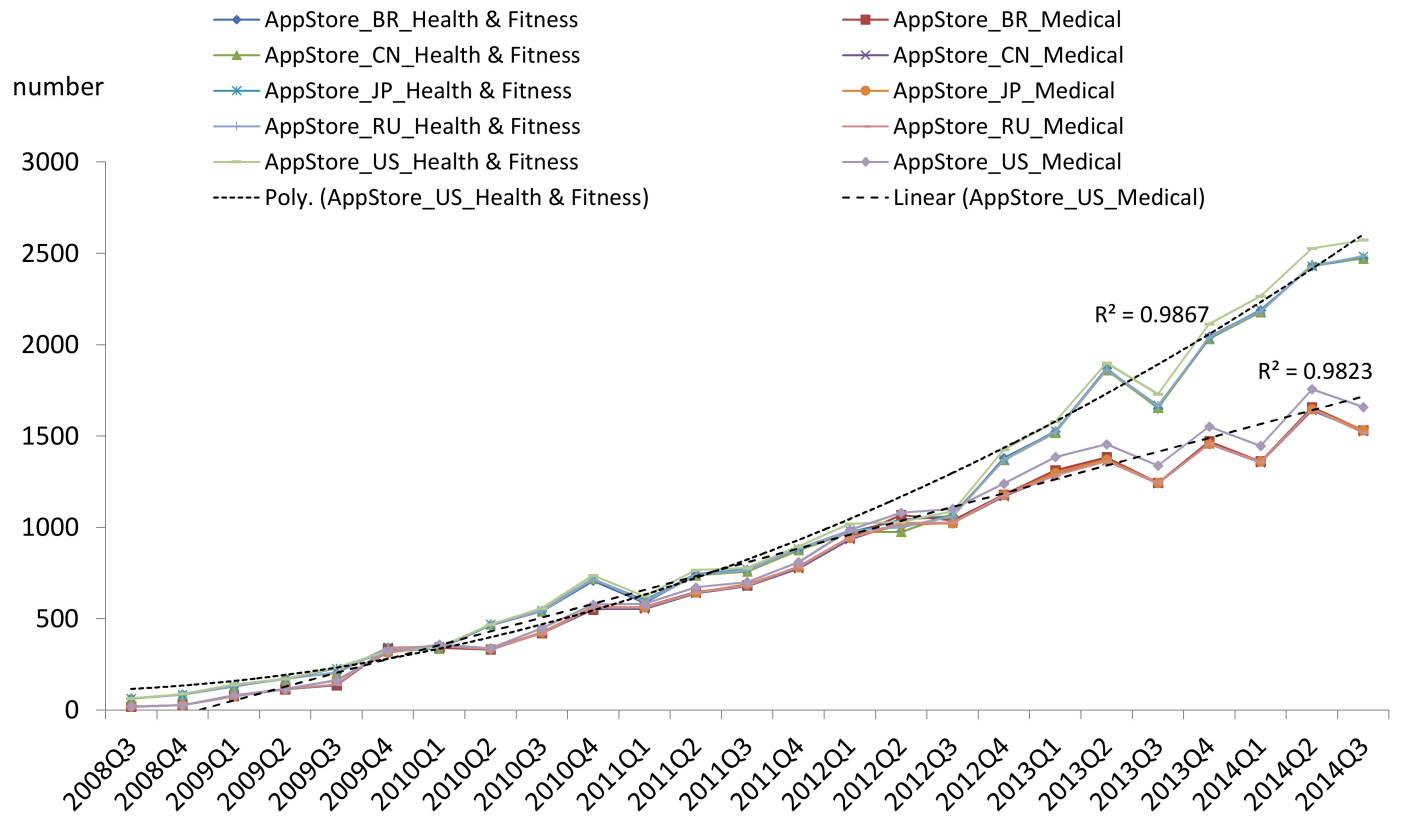
The trend of the number of released mHealth apps in the Apple App Store (AppStore). 2008Q3: third quarter of year 2008. BR: Brazil; CN: China; JP: Japan; RU: Russia; US: United States.

Based on the release date information of each app included in our repository, we can analyze the trend of mHealth apps available in the AppStore. We plotted the number of apps released in each quarter since the third quarter of 2008 (Figure 1 shows this). From this figure, we can see that the apps in the Health & Fitness category show a quadratic growth (R^2^ = 0.9867), while the apps in the Medical category demonstrate a linear growth (R^2^ = 0.9823). The patterns in the top five countries are similar for both the Health & Fitness and Medical subcategories. The GooglePlay doesn’t contain the released date information of apps; instead, only the updated date information is available. More than 76.57% (9397/12,272) of the apps were updated in the last two quarters. Therefore, the trend for apps in GooglePlay was not analyzed in this study.

## Discussion

### The mHealth App Repository

The mHealthApps repository allows us to analyze thousands of apps in the market systematically and efficiently, and can be utilized to provide an overview of the trends for mHealth apps. The repository is scheduled to be updated quarterly. Detailed information of all these apps can be freely requested from the repository website [18], but will be restricted for personal and noncommercial use only. A unique feature of our repository is that it provides a new dimension of information of apps, such as the user behavior, which is neglected by many other studies in the field. The user behavior data, including the average user rating, the number of user ratings received per app, and the distribution of user ratings in the five-star rating system are based on millions of mHealth apps users worldwide, and have been tested on the real market. The repository also contains other information, such as the price, the released/updated date, and the app descriptions, which can be used for further business marketing, activity analysis, detail subcategories decomposition, and so on.

### Limitations

It is noted that our study has some limitations. First, the category of each app is submitted by the app’s owner and approved by the app store. Therefore, the accuracy of app categorization is beyond our control. Additional strategy based on nature language processing would be necessary to ensure all the apps included in our repository are health-related. Second, we only retrieved mHealth apps from the two most established system platforms, the iOS (AppStore) and the Android (GooglePlay), there are also apps from other platforms, such as the Windows Phone Store [20] and the BlackBerry World [21]. Third, our repository is limited in the regions the information was extracted from. For the AppStore, we only extracted apps information from the top 5 regions according to the market size, which neglects information from other well developed countries such as Australia and European countries (different stores are separated by different languages), as well as from fast developing regions such as Africa and India. For the Android platform, we only extracted apps information from the GooglePlay US store, due to the complex Android markets in other countries. For example, in China, the major Android stores include Baidu Shouji Zhushou [22], Tencent Yingyongbao [23], and 360 Shouji Zhushou [24], while the GooglePlay is not among the major Android stores. Fourth, the number of apps from the GooglePlay is limited due to the availability of apps on the GooglePlay website, which only lists up to 600 of the most popular apps every day. Our repository is based on the lists of apps accumulated between July 24 and December 6, 2014. In spite of these limitations, we expect this mHealth app repository will not only serve as a centralized information resource for researchers to perform meta-analysis on current apps, but also provide guidance for future research designs in the mHealth field.

## Multimedia Appendix 1

Zip flat file with array format for AppStore Health & Fitness apps in United States.

## Multimedia Appendix 2

Zip flat file with array format for AppStore Health & Fitness apps in China.

## Multimedia Appendix 3

Zip flat file with array format for AppStore Health & Fitness apps in Japan.

## Multimedia Appendix 4

Zip flat file with array format for AppStore Health & Fitness apps in Brazil.

## Multimedia Appendix 5

Zip flat file with array format for AppStore Health & Fitness apps in Russia.

## Multimedia Appendix 6

Zip flat file with array format for AppStore Medical apps in United States.

## Multimedia Appendix 7

Zip flat file with array format for AppStore Medical apps in China.

## Multimedia Appendix 8

Zip flat file with array format for AppStore Medical apps in Japan.

## Multimedia Appendix 9

Zip flat file with array format for AppStore Medical apps in Brazil.

## Multimedia Appendix 10

Zip flat file with array format for AppStore Medical apps in Russia.

## Multimedia Appendix 11

Zip flat file with array format for GooglePlay Health & Fitness apps in United States.

## Multimedia Appendix 12

Zip flat file with array format for GooglePlay Medical apps in United States.

## Multimedia Appendix 13

Disclaimer.

## Abbreviations

API: app program interface
apps: apps
AppStore: Apple App Store
BR: Brazil
CN: China
GooglePlay: Google Play Store
ID: identity
JP: Japan
JSON: JavaScript Object Notation
mHealth: mobile health
RU: Russia
